# Co-Designing a New Yoga-Based Mindfulness Intervention for Survivors of Stroke: A Formative Evaluation

**DOI:** 10.3390/neurolint14010001

**Published:** 2021-12-21

**Authors:** Tharshanah Thayabaranathan, Maarten A. Immink, Susan Hillier, Rene Stolwyk, Nadine E. Andrew, Philip Stevens, Monique F. Kilkenny, Emma Gee, Leeanne Carey, Amy Brodtmann, Julie Bernhardt, Amanda G. Thrift, Dominique A. Cadilhac

**Affiliations:** 1 School of Clinical Sciences at Monash Health, Monash University, Clayton, VIC 3168, Australia; monique.kilkenny@monash.edu (M.F.K.); amanda.thrift@monash.edu (A.G.T.); dominique.cadilhac@monash.edu (D.A.C.); 2 Centre of Research Excellence in Stroke Rehabilitation and Brain Recovery, Heidelberg, VIC 3084, Australia; rene.stolwyk@monash.edu (R.S.); nadine.andrew@monash.edu (N.E.A.); lcarey@unimelb.edu.au (L.C.); agbrod@unimelb.edu.au (A.B.); Julie.Bernhardt@florey.edu.au (J.B.); 3 College of Nursing and Health Sciences, Flinders University, Adelaide, SA 5042, Australia; maarten.immink@flinders.edu.au; 4 IIMPACT, Allied Health and Human Performance, University of South Australia, Adelaide, SA 5000, Australia; Susan.Hillier@unisa.edu.au; 5 Turner Institute for Brain and Mental Health, Monash University, Clayton, VIC 3800, Australia; 6 Peninsula Clinical School, Monash University, Frankston, VIC 3199, Australia; 7 Consultant, Mooreville, TAS 7321, Australia; info@chiasma.info; 8 Stroke Division, The Florey Institute of Neuroscience and Mental Health, Heidelberg, VIC 3052, Australia; 9 Survivor of Stroke, Inspirational and Motivational Speaker, Cotham, VIC 3101, Australia; emma@emma-gee.com

**Keywords:** stroke, evaluation research, qualitative evaluation, stakeholder engagement, co-design, community-based intervention

## Abstract

Movement-based mindfulness interventions (MBI) are complex, multi-component interventions for which the design process is rarely reported. For people with stroke, emerging evidence suggests benefits, but mainstream programs are generally unsuitable. We aimed to describe the processes involved and to conduct a formative evaluation of the development of a novel yoga-based MBI designed for survivors of stroke. We used the Medical Research Council complex interventions framework and principles of co-design. We purposefully approached health professionals and consumers to establish an advisory committee for developing the intervention. Members collaborated and iteratively reviewed the design and content of the program, formatted into a training manual. Four external yoga teachers independently reviewed the program. Formative evaluation included review of multiple data sources and documentation (e.g., formal meeting minutes, focus group discussions, researcher observations). The data were synthesized using inductive thematic analysis. Three broad themes emerged: (a) MBI content and terminology; (b) manual design and readability; and (c) barriers and enablers to deliver the intervention. Various perspectives and feedback on essential components guided finalizing the program. The design phase of a novel yoga-based MBI was strengthened by interdisciplinary, consumer contributions and peer review. The 12-week intervention is ready for testing among survivors of stroke.

## 1. Introduction

Stroke is a leading cause of death and disability in Australia, with survivors often experiencing problems with physical, cognitive, and psychological health [1]. There are very few community-based intervention options available for long-term management and potential recovery following stroke. There is emerging evidence on the potential benefits of movement-based mindfulness interventions (MBI), such as yoga, as an adjunct therapy to mitigating the influence of risk factors, such as high blood pressure, cholesterol, and problems with anxiety or depression [2,3]. MBIs are complex interventions with multiple components, including static body postures and slow, effortful dynamic movements combined with breathing techniques and meditation [2].

Extensive planning and formative research are an essential phase in the successful development and implementation of a new intervention that might be tested for its efficacy in a randomized controlled trial (RCT). This is particularly important when the intervention is complex, because it has multiple components or mechanisms of action. A pilot study is an essential preliminary phase of an RCT for testing the effectiveness of a newly developed intervention [4]. However, if the new intervention to be tested is complex (i.e., has multiple components, or where there are several mechanistic pathways) [5], it must be designed carefully and systematically across different developmental stages to ensure standardization for application and reproducibility.

In this article, we aim to describe the processes involved with co-designing an intervention, and the formative evaluation that was undertaken to inform the final design of this new, contextually appropriate yoga-based MBI for survivors of stroke, which will be tested in a future phase II RCT.

## 2. Materials and Methods

### 2.1. Study Design

**Figure 1:**
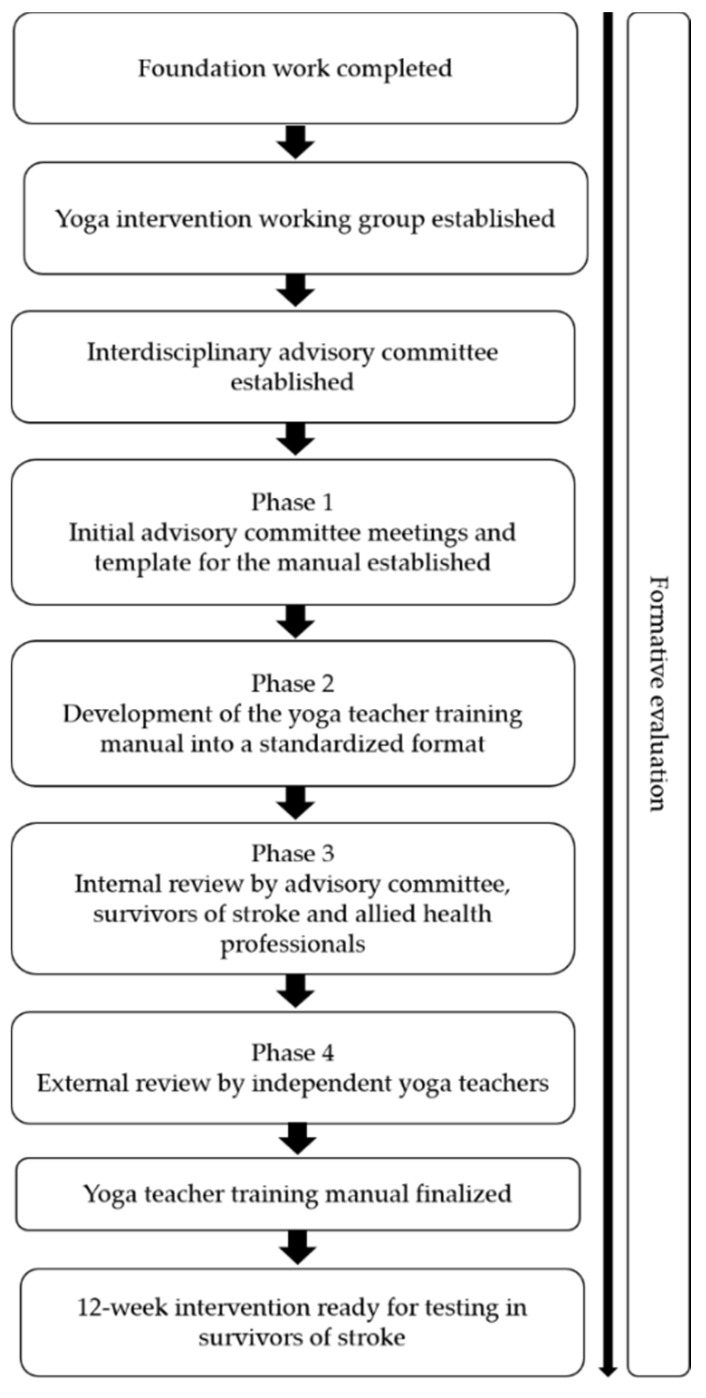
Summary of the processes.

This project was undertaken in two main parts as outlined below. The intervention was developed over two years (June 2016–June 2018), with formative evaluation iteratively conducted during the development period. Multiple data sources were used to document the processes involved with designing and developing the intervention (Figure 1).

### 2.2. Part A: Literature Review and Establishment of Advisory Committee

#### 2.2.1. Literature Review

Foundation work including a broad literature review was undertaken. We identified one existing yoga program for stroke that had been used in Australia, which was trialed as a 10-week intervention [6]. The authors who designed this intervention had concluded that it was acceptable and provided evidence of improvements in mental health and quality of life [6], and perceived meaningful functional, psychological, and social benefits [7]. However, only patients with chronic hemiparesis with minimum levels of physical ability (i.e., ability to walk for 10 m or more with or without the use of an assistive walking device) were recruited for investigation of this previous yoga for stroke program, potentially limiting the generalizability of the intervention. Furthermore, the previous yoga for stroke program was developed without consultation from stakeholders, and was not based on any development framework.

#### 2.2.2. Advisory Committee

Investigators (M.A.I. and S.H.) of the previous yoga for stroke program undertaken in South Australia were invited to collaborate to enhance the design of the current yoga program (yoga intervention working group). We then established an interdisciplinary advisory committee to design the intervention and future clinical trial. T.T. and D.A.C. were tasked with project coordination and collection and synthesis of data as part of the formative evaluation process. To partner in the development process, stakeholders (i.e., survivors of stroke and a carer) were invited and their informed consent was obtained.

### 2.3. Part B Yoga Program Intervention Development

We used the Medical Research Council complex interventions (MRC) framework to develop the intervention [8,9,10]. We undertook this process, which involves exploring relevant theory and synthesizing the existing evidence, to support decision making about the intervention design. In this part, the yoga-based MBI was developed in the format of a yoga teacher’s training manual over four main phases, whereby the formative evaluation data were used to inform each subsequent phase (Table 1).

### 2.4. Formative Evaluation

#### 2.4.1. Data Collection and Documentation

Qualitative data were sourced (i.e., included notes from advisory committee and working group meetings, as well as focus groups with yoga teachers) to describe the development of the training manual by T.T. and D.A.C. Documentation analysis included: (1) email and phone correspondence between members of the working group and key members of the advisory committee, (2) comments on hard copy drafts of the manual from consumers and yoga teachers, (3) minutes of all advisory committee and working group meetings, and (4) notes from a researcher’s observations (T.T.). Edits to the manual were tracked into a copy of the master document by all the different contributors. The master document was consolidated to ensure all relevant feedback was incorporated from each phase of the development process.

#### 2.4.2. Data Synthesis and Interpretation

Synthesis of the feedback and, where relevant, pragmatic inductive thematic analysis techniques were used. Identified themes were collated and compared. Core intervention and manual content features that were required to be modified were discussed and amended, as required, within the members of the yoga intervention working group. Similarly, if there was mixed or conflicting advice, this was adjudicated by the members of the yoga intervention working group.

## 3. Results

Across the four phases of yoga program intervention development (as described above in Part B), three broad themes, and related sub-themes, emerged from the thematic analysis: (1) MBI content and terminologies (e.g., intervention duration, guidance on transition to home practice, use of consistent terminologies); (2) manual design and readability (e.g., photos to aid postures and transitions); and (3) barriers and enablers to delivering the intervention (e.g., class locations, support equipment). The processes involved, themes, and sub-themes in each phase are presented below.

### 3.1. Phase 1: Establishment of Advisory Committee to Co-Design the Program and Establish the Template for the Manual

For Phase 1, an advisory committee was established that included experts from various fields, including a clinical neuropsychologist, epidemiologist, statistician, exercise physiologist, occupational therapists, physiotherapists, yoga experts, basic scientists, and a neuroscientist. Survivors of stroke (*n* = 3) and a carer were invited to be members of the advisory committee and to contribute to the design and development process.

The agreed first aim of Phase 1 was to design a template of the yoga teacher training manual. During this first advisory committee meeting, results on the relative theories, synthesized evidence, and a summary of the previous 10-week intervention were presented to support decision making [6,11]. The advisory committee then primarily discussed the study design, the length of the program, inclusion criteria, involvement of caregivers, possible outcome measures, and strategies to maximize retention (e.g., accessibility to group classes). The advisory committee agreed to not duplicate efforts, but to improve on the previously developed 10-week program, which was shown to be feasible in its trial. It was also agreed that the program should be a 12-week intervention to allow sufficient time for the reinforcement of MBI techniques and transition to home practices.

Phase 1 assisted in maintaining fruitful and open communication between the research team and stakeholders (i.e., survivors of stroke, carer, and yoga teachers) via regular emails and telephone calls. This phase also fostered a good working relationship between the stakeholders, researchers, and clinicians. The stakeholders had a critical role in supporting other members of the advisory committee to understand factors that needed consideration for the design of the program. This included the structure of the intervention, accessibility and size of classes, influence of group participation, and involvement of caregivers in classes. The consumers provided information on the various benefits and difficulties of participating in standard (generic) yoga classes from their perspective, i.e., what had and had not helped with their participation in generic yoga programs in the community, and in their recovery. This review process ensured the manual-based program would be practical, achievable, and inclusive for participants of varying abilities.

Formative evaluation: In this phase, three major themes and four sub-themes were revealed that led to specific decisions being made in relation to the format of the yoga program. These are detailed below.

#### 3.1.1. Theme 1: Length and Content of Program

Following the initial decision to develop a 12-week program, the following three decisions were made related to the development of the intervention. Firstly, the intervention should be comprised of 12 different group classes progressing in intensity and duration over the course of the program. Secondly, the first class would be needed to help participants understand the objectives of the program and to familiarize them with the poses and transitions. Lastly, the final weeks (weeks 11 and 12) would be used to reinforce the various aspects of the program and provide tips to safely practice at home. Adding daily home practices between classes was also discussed. It was also decided that there would be rolling start dates for participants. Therefore, the teachers would offer a range of options for participating in the techniques based on a participant’s class timeline, their movement abilities, and developing competency at directing attention to the movement, breathing, or meditation.

#### 3.1.2. Theme 2: Assistant Teachers

Based on the experience of the previous 10-week program [6,7], the advisory committee agreed on having an assistant teacher present at every class. This was to ensure the lead yoga teacher could teach without interruption and the assistant could help correct the poses of participants, or to provide support in transitioning safely between poses, as required.

#### 3.1.3. Theme 3: Template Design and Sub-Themes

##### Sub-Theme 1: Background Information on Stroke for Teachers

A decision was made to include in the manual information on stroke, major risk factors for stroke, and consequences that may occur after stroke. This section would inform those yoga teachers and class assistants who may have limited understanding about stroke and its consequences. This section had not been part of the prior 10-week program, but was considered important if a larger number of yoga teachers were to be trained to deliver the program.

##### Sub-Theme 2: Mindfulness Development

There was consensus that the active ingredient of the yoga program for stroke as an intervention was the development and attainment of focused attention through mindfulness mediation, as well as the use of movement via the various poses and concentration on breathing to focus the mind. Consistent with a standard yoga class structure (movement, focus on breathing throughout the class, and meditation), the last part of each group class would have at least 10 min dedicated to mindfulness meditation only. The focus was to ensure the purposeful allocation of attention so as to have full awareness of experiences in a non-judgmental and accepting manner [12]. It was decided to allow at least 10 min for formal mindfulness meditation. This would be increased to 20 min by the last session.

##### Sub-Theme 3: Clear Illustration of Poses

Survivors of stroke with different levels of impairment were invited for a photo shoot to capture yoga poses that would be included in the manual. The photos were required to provide clear illustrations of how the poses should be completed safely and, as relevant, with stable support furniture (e.g., chair without wheels).

##### Sub-Theme 4: Active Therapy Feature

Importantly, the yoga and allied health experts agreed that the manual must highlight the concept “where participants with stroke, particularly those with physical disabilities, are encouraged to use visual imagery of themselves moving the non-functioning limb”. This gives the patient the illusion of moving the non-functioning limb, a process which is proposed to stimulate and reorganize function in damaged neural networks of the adult brain [13]. Another option is to sometimes use the active arm to physically assist the weaker arm by holding hands and then raising both arms up above in a bilaterally symmetrical movement.

### 3.2. Phase 2: Development of the Yoga Teacher Training Manual to Detail the Intervention Components and Features in a Standardized Format by the Working Group

The main aim of Phase 2 was to formulate the draft manual based on the information gathered in Phase 1. Three major themes were identified in this phase.

#### 3.2.1. Theme 1: Yoga Intervention Working Group

The first step involved development of the terms of the reference document highlighting the roles and responsibilities of the members of the working group. The second step involved the development and execution of a collaborative agreement between members of the working group.

#### 3.2.2. Theme 2: Program Format and Content

The program manual included details of the role of the teacher in ensuring program fidelity. The main headings were agreed upon by the working group, and T.T. wrote the initial draft for many of the subsections. Members of the advisory committee with expertise in stroke contributed feedback. To ensure all yoga teachers would understand the underlying principles of the program, it was agreed to have a section outlining a set of ‘golden rules’ (i.e., guiding principles) of the main program features that must be adhered to throughout program. Group classes were designed to be delivered by an experienced yoga teacher weekly for one-hour sessions over 12 weeks. A description of the various physical postures, possible variations to the postures, and suggestions on appropriate use of language (i.e., avoid Sanskrit terminologies) were included in the manual.

#### 3.2.3. Theme 3: Photo Shoot

The two survivors of stroke who were part of the advisory committee, and another, were invited to take part as models in a photo shoot. There were two females and one male, with different levels of abilities, who attended the photo shoot. The photos captured were used to illustrate some of the poses included in the manual. At the beginning of the shoot, the models were provided with an information sheet. The shoot was led by M.A.I., while T.T. observed the photo shoot and documented the importance of safe transition from one pose to another. This was important especially for someone with severe physical disability who would require a strong and secure chair for support, or modified means of support in getting up and down from the ground (e.g., using the support of a wall or receiving help from an assistant). The models also informed M.A.I. and T.T. that they had been practicing yoga and mindfulness meditation for a long time (>3 years) through generic programs in the community. All models expressed pleasure in being a part of project, and were excited that a tailored intervention was being developed for use in survivors of stroke.

### 3.3. Phase 3: Internal Review of the Program by Members of the Advisory Committee and Other Consumers and Allied Health Professionals

All members of the working group provided their expert opinion on the content and design of the manual. In addition, through purposeful sampling, allied health professionals (who were also stroke experts, *n* = 3) and people experiencing a stroke and a carer (*n* = 3) were invited to collaborate and review the content and design of the drafted prototype manual (as described in Phase 2). Members of the advisory committee were also asked to provide their view on the content, design, overall acceptability, and feasibility of the content in the manual. This process lasted seven months (from January 2017 to August 2017). Four major themes were identified in this phase.

#### 3.3.1. Theme 1: Refining Manual Format and Content

Through written feedback, the allied health professionals and researchers commented on how particular activities could be better adapted to suit survivors of stroke, whilst maintaining the guiding principles of the program. Examples include:

*Advisor 1: “Empower participants to participate at a level that they are comfortable and may include sitting on a chair in some or all exercises. Safety within a class and a home must be carefully thought about (e.g., type of chairs, used, floor surfaces, tripping and other hazards)”.*

*Advisor 2: “Are participant able to do this pose with assistance from a caregiver. If so, this needs to be stated and the role of caregiver within the intervention defined”.*

*Advisor 3: “Avoiding injuring, safety is the priority. Imagine both limbs doing it, and encourage participant to use imagery technique”.*

#### 3.3.2. Theme 2: Transitioning to Home Practice

It was also suggested that the content of week 11 and week 12 could largely focus on ‘transition to home techniques’ and include information on how to effectively continue practice at home, rather than traditional group classes. The manual was further modified to incorporate the suggestions provided by the advisory committee and stakeholders. Changes in the class plans were also made to (1) facilitate ease of implementation for teachers, (2) use materials that were available and acceptable for the cohort, and (3) promote implementation fidelity of the program. Practice points were embedded under sections of each posture and class plans to reinforce the major guiding principles of the program. It was also reinforced that an assistant teacher must be present within each class to optimize program delivery and patient safety.

#### 3.3.3. Theme 3: Clear Instructions on Transitions

Importantly, greater detail was added on how to transition from one pose to another. Photos that provided an illustration of some of the poses were also updated to include more contextually appropriate photographs (e.g., avoid ones with roller chairs, encourage use of pillows for support).

#### 3.3.4. Theme 4: Story of the Three Models

The manual was personalized by adding the ‘story’ of the 3 models who had contributed to the photo shoot at the end of the manual. It was believed that, by including these stories, the yoga teachers undergoing training would gain some insights into what it is like to live with stroke and participate in yoga.

### 3.4. Phase 4: External Review of the Training Manual by Independent Yoga Teachers, and Finalization of Content

Through the collaborative network of D.A.C., and via email to the Yoga Australia Association, four yoga teachers (3 females, 1 male) not previously involved in the project were approached to provide independent review of the manual. These yoga teachers had different levels of experience and provided their expert opinion on the content, design and acceptability of the drafted manual over a four-month period (August to December 2017). The yoga teachers also attended a focus group and trial training workshop in December 2017 where they provided additional feedback on the manual. Several aspects of the manual (e.g., section on stroke) were deemed feasible and appropriate.

Some modifications were identified that needed to be made to the manual to enhance its relevance and feasibility for use in program delivery, for example, including additional instructions to support people with disabilities while sitting on the floor, adding key limitations section in dot points under each pose, and providing alternative seating options or transitions for those unable to move from standing to floor quickly.

In this phase, three major themes were identified.

#### 3.4.1. Theme 1: Use of Consistent Terminologies

The teachers indicated that it would be beneficial to focus on the main concepts of the program and the use of consistent language prior to delivering the first class. Clear terminologies (western or plain English) were suggested to be used instead of Sanskrit words, such as using ‘deep controlled breathing’ instead of ‘pranayama’ for better readability.

#### 3.4.2. Theme 2: Alterative Seating Options

The teachers suggested including alternative seating options. For example, the use of chairs instead of standing for any ‘standing pose’ could easily be implemented in the group environment. Therefore, this type of practical information was included, where relevant.

#### 3.4.3. Theme 3: Further Refinement to Other Manual Content

Teachers also provided information about the suitability of particular resources used in the classes, for example yoga bands for support of shoulder or arm poses. They also advised that the manual should include how to address any concerns about safety and adverse events during class times. Finally, the teachers suggested adding information on the different etiology of stroke to the background section on stroke.

In response to the independent review outlined above, we modified the yoga teacher training manual. Overall, positive feedback was received from the allied health professionals, yoga teachers, and consumers about the program and its potential utility. This manual has been designed to train and then be used by yoga teachers to teach groups of survivors of stroke across a broad range of age groups and disabilities in a Phase II trial. (i.e., Phase II of the MRC framework).

## 4. Discussion

In this study, we provide a description of a systematic, sequential approach to integrating scientific evidence from systematic reviews, qualitative research, and expert knowledge, as well as knowledge and experience with stakeholders to develop an evidence-based complex intervention. We have described in detail how co-design and formative evaluation strategies helped inform the recommendations and refinement of the yoga-based MBI for survivors of stroke with varying abilities who live in the community.

This study, to the best of our knowledge, is the first one to document the development of a complex yoga-based MBI for stroke using methodology prescribed by the MRC framework. Before a complex intervention is piloted in a large clinical trial and tested for its effectiveness, it is important that it is designed well to ensure relevance and the ability to be standardized and replicated [14,15]. As a result of the evaluation, a number of implementation issues were raised, and modifications made accordingly to improve content and delivery of the intervention. The developed intervention will be used to train teachers who will be delivering the intervention in Phase II of the MRC framework (i.e., as an exploratory trial).

Formative evaluation is an essential feature of the development and design phase of a new multiple-component intervention or program. The focus of formative evaluation is to uncover the advantages and disadvantages of the intervention during its development process with the purpose of generating evidence to improving the intervention features so that it is implemented as intended, and will produce the hypothesized benefits for the patient group to which it will be applied [16]. Results of the formative evaluation provided a firm basis for improving the prototype of the intervention towards a high-quality and completed intervention, as well as sharpening the underlying design principles towards a final set of design principles [17].

The involvement of people who had experienced a stroke and a carer in the development stage and other stages of clinical trials is encouraged [18,19]. In particular, engagement of these key research partners in the planning, adaptation, and modification of the intervention was reliant on their early involvement in the project and facilitated a strong design for testing the program in a future clinical trial [20]. In this study, these stakeholders provided mutually valuable contributions, via regular meetings, emails, and focus group, and were regarded as equal and active members, and not merely as passive subjects or recipients of services. Moreover, the suggestions made by these stakeholders (i.e., survivors of stroke, carer, and yoga teachers) informed important practical modifications required to increase program adherence for survivors of stroke with varying disabilities.

A major strength of our study is the ‘co-design’ approach whereby a team of allied health professionals, researchers, yoga experts and teachers, survivors of stroke, and a carer worked together [21]. The multidisciplinary team was essential to identify features to include, barriers to program implementation, and intervention delivery. This collaborative and phased approach was invaluable for identifying various aspects of the manual that needed to be modified to ensure relevance to the target population. At face validity, the developed intervention was deemed acceptable and suitable for people with stroke and all types of impairments.

## 5. Conclusions

This study provides a model for other researchers and clinicians who aim to develop and implement complex interventions for survivors of stroke in the community. Illustrating how the yoga-based MBI for stroke was adapted and successfully integrated into a training manual for pilot testing through the processes of co-design provides insights into how this may be accomplished in similar contexts and other populations with chronic disabilities. A number of lessons learned throughout the evaluation process helped further improve the intervention.

Ensuring that the intervention is clearly detailed will allow it to be replicated if found to be effective. Effective implementation and trialing of this program is essential for delivering robust evidence about the efficacy of a yoga-based MBI in survivors of stroke. The finalized intervention is to be tested for effectiveness in a future Phase II trial and, subsequently, in a larger Phase III RCT, aimed at assessing the impact of the yoga-based MBI on patient outcomes, including quality of life and mental health disorders in survivors of stroke living in the community.

## Figures and Tables

**Table 1:** The program was developed over four main phases.

Phases	Description
Phase 1	Initial advisory committee meetings to achieve consensus on the broad principles of the intervention scope and establish the template for the manual
Phase 2	Development of the yoga teacher training manual to detail the intervention components and features in a standardized format
Phase 3	Internal review of the program by members of the advisory committee and other stakeholders (i.e., survivors of stroke) and allied health professionals
Phase 4	External review of the training manual by independent yoga teachers, and finalization of content

## Data Availability

No new data were created or analyzed in this study. Data sharing is not applicable to this article.
